# Toll-like receptor 4 (TLR-4) polymorphisms and asthma risk in rural and urban settings: findings from the UK biobank

**DOI:** 10.48101/ujms.v130.12243

**Published:** 2025-07-04

**Authors:** Marta A. Kisiel, Mathias Rask-Andersen, Åsa Johansson, Weronica E. Ek, Anna Rask-Andersen

**Affiliations:** a Occupational and Environmental Medicine, Department of Medical Sciences, Uppsala University, Uppsala, Sweden; b Department of Immunology, Genetics and Pathology, Science for Life laboratory, Uppsala University, Uppsala, Sweden

**Keywords:** Polymorphisms within the *TLR4*gene, asthma, residential area such as rural versus urban, early/late onset asthma, asthma with allergy

## Abstract

**Introduction and aim:**

The risk of asthma and its phenotypes may be modified by gene-environmental interactions. The previous studies on the interactions between genetic variations in the toll like 4 (TLR4)*,* the main receptor for bacterial endotoxin, and asthma were contradictory as they were underpowered and did not consider different asthma phenotypes. The main aim of this study was to identify interactions between two single nucleotide polymorphisms (SNPs) within the *TLR4* gene, Asp299Gly and Thr399Ile, and residential area (urban or rural) in females and males with asthma and different asthma phenotypes.

**Method:**

This study was performed on 38,332 asthmatics and 322,852 non-asthma (both British Caucasians) subjects from the UK Biobank database. Asthma was also divided into phenotypes, such as asthma with/without allergy and early/late onset asthma. The residential area was based on the population area density and classified as urban or rural living. Multivariate regression models adjusted for age, body mass index, and smoking status were used to analyze interactions between the SNPs, residential area in asthma, and asthma phenotypes. The association between asthma and residential area or the SNPs was also determined.

**Result:**

There were no significant associations between the SNPs and asthma risk (for Asp299Gly: OR (95% CI): 1.00 (0.97–1.02), for Thr399Ile: 0.99 (0.96–1.02) or between the SNPs and asthma phenotypes in either sex or combined cohorts. The effects of the SNPs were not modified by residential area population density in either sex with asthma or across asthma phenotypes. Asthma and its phenotypes were not associated with the SNPs or residential area.

**Conclusions:**

Our study found no statistically significant association between *TLR4* polymorphisms and asthma, regardless of sex or residential area. Further studies are needed to clarify the functional impact of TLR4 variation in asthma pathophysiology.

## Introduction

Asthma is a chronic and heterogeneous respiratory disease with increasing global prevalence (1). It is characterized by persistent airway inflammation, bronchial hyperresponsiveness, and reversible airflow obstruction, and manifests in diverse phenotypes driven by complex interactions between genetic, environmental, and epigenetic factors (2). These phenotypes underscore the heterogeneity of asthma and reflect its multifactorial origins, making it challenging to identify the specific factors contributing to its development (3, 4).

One key environmental factor associated with asthma risk is microbial exposure, particularly to endotoxin, a potent inflammatory molecule derived from bacterial lipopolysaccharides (LPS), a component of gram-negative bacterial cell walls. LPS trigger innate immune responses and can have protective and detrimental effects on asthma development (5). According to the ‘Hygiene hypothesis’, frequent exposure to microbial compounds like endotoxin during early immune development confers protection against asthma (6, 7) by promoting a Th1-dominant immune response rather than a Th2-driven phenotype linked to atopy and asthma (4). Rural environments typically expose individuals to higher microbial loads, including LPS, through contact with animals, soil, and plants (8). This increased microbial exposure is thought to protect against asthma, particularly allergic asthma (9). In contrast, urban living is associated with higher asthma prevalence, particularly in children, due to reduced microbial exposure, greater allergen and irritant exposure, and prolonged time indoors with higher levels of indoor allergens (8). However, evidence from studies on bacterial colonization in the upper respiratory tract of infants suggests that certain microbial exposures may increase asthma risk early in life (10), and indicate additional genetic factors to modulate this effect (11).

Toll-like receptor 4 (TLR4) plays a key role in asthma pathogenesis as a pattern recognition receptor (PRR) involved in detecting LPS triggering, innate immune, and inflammatory responses (12, 13). Genetic polymorphisms in the TLR4 gene, particularly rs4986790 and rs4986791, have been studied for their potential roles in modulating asthma susceptibility (14). These single-nucleotide polymorphisms (SNPs) are located within exon three of TLR4, and lead to the amino acid substitutions: Asp299Gly and Ile399Thr (11).

Earlier studies have suggested that these SNPs were associated with a reduced inflammatory response to LPS, potentially lowering asthma risk (15). However, findings have been inconsistent, with some studies reporting a protective effect, while others found no significant association between these SNPs and asthma or allergic conditions (16–21). Notably, many studies were limited by small sample sizes and did not account for key environmental exposures that may influence the effects of TLR4 on asthma susceptibility (14).

Given the inconsistencies in the existing literature, this study aims to explore the interaction between genetic predisposition and environmental exposures in asthma susceptibility. Understanding these interactions could contribute to the development of personalized prevention and treatment strategies. Using residential living environments (rural vs. urban) as a proxy for microbial exposure, we hypothesize that TLR4 polymorphisms (Asp299Gly and Thr399Ile) may modulate asthma risk differently depending on the living environment. Specifically, individuals carrying these polymorphisms may experience a reduced asthma risk in rural settings, where higher endotoxin exposure could influence immune tolerance. Conversely, individuals without these polymorphisms may derive greater protective benefits from rural microbial exposure. At the same time, those living in urban environments may have a higher asthma risk due to lower microbial diversity and increased allergen exposure.

The primary objective of this study was to evaluate the association between TLR4 polymorphisms, residential environment, and asthma risk. In addition, we examine whether these effects vary by asthma phenotype (allergic vs. non-allergic), age of onset (early vs. late), and sex.

## Materials and methods

### Study design

This study utilized data from the UK Biobank, a large population-based prospective cohort study, comprising 502,682 adults (aged 37–73 years at recruitment between 2006 and 2010) from the United Kingdom (22). The UK Biobank contains in-depth genetic information, which includes the polymorphisms within TLR4: rs4986790 (Asp299Gly) and rs4986791 (Thr399Ile), as well as anthropometric and lifestyle data collected via touch questionnaire.

Genotyping was performed on two high-density arrays: the UK BiLEVE and Axiom arrays. For this study, we included participants with available genotyping who self-identified as British Caucasian to avoid any effects of population stratification. Additionally, individuals whose self-reported sex differed from their genetically determined sex were excluded from the analysis. Genotyping and QC procedures for the UK Biobank have been described elsewhere (23).

### Definitions

Asthma (data field 6152) was an affirmative response to the question: ‘Has a doctor ever told you that you have had asthma?’ Allergy (data field 6152) was a positive response to the question: ‘Has a doctor ever told you that you have had hay fever, allergic rhinitis, or eczema?’ Asthma with allergy was affirmative responses to questions on asthma and allergy.

Early- and late-onset asthma were determined based on the self-reported age at asthma diagnosis (data field 22147**)**. Early-onset asthma was defined as a diagnosis occurring at or before 13 years of age, while late-onset asthma was defined as a diagnosis after 13 years. The cutoff of 13 years was selected in accordance with a previous meta-analysis (24).

Residential area population density (data field 20118) in units as individual per km^2^ was classified as rural or urban based on post-code derived population density. A rural area was defined as a settlement of 10,000 or fewer inhabitants in England and Wales and 3,000 or fewer inhabitants in Scotland, following classifications from the Office for National Statistics (ONS, 2013) (25). Areas exceeding these population thresholds were classified as urban. This classification was generated from the UK 2001 census (http://geoconvert.mimas.ac.uk/index.htm).

Body mass index (BMI) in kg/m^2^ (data field 21001) was calculated from weight in kilograms divided by height squared (kg/m^2^), using measurements obtained during the participant’s baseline visit at UK Biobank assessment centers. Smoking status was determined from questionnaire data (data field 20116) and was classified as never smokers (individuals reported having never smoked) and ever smokers (who reported current or past smoking).

### Statistical methods

Multivariable logistic regression models, adjusted for age, BMI, and smoking status, were used to assess the association between residential area (rural vs. urban) and asthma in males and females. Subsequently, additional multivariable logistic regression models, further adjusted for residential area, were used to evaluate the association between TLR4 polymorphisms (Asp299Gly and Thr399Ile) and asthma or its phenotypes. Results were reported as odds ratios (OR) with 95% confidence intervals (95% CI).

Multivariable logistic regression models were used to assess interactions between rs4986790 (Asp299Gly), rs4986791 (Thr399Ile), residential area (urban vs. rural), and asthma status (asthma vs. non-asthma), as well as asthma phenotypes (asthma with allergy vs. asthma without allergy; early vs. late-onset asthma) in males and females.

To control for potential confounding effects, statistical models were adjusted for age, BMI, smoking status, and 10 principal components to account for population structure. Interaction models included secondary interactions between all covariates (26). *P*-values were calculated using Student’s t-test, with *P* < 0.05 considered statistically significant. All analyses were conducted using R v3.6.0 and PLINK v1.90b3.32 (27).

### Ethical statement

Access to the UK Biobank genetic and phenotypic data was granted under application no. 15479: ‘Genetic architecture of immune diseases and allergies’. The analyses conducted in this study were also approved by the Swedish ethical review authority (registration number: 2020-04415).

The UK Biobank possesses a generic Research Tissue Bank approval granted by the National Research Ethics Service (http://www.hra.nhs.uk/), which permits researchers to use UK Biobank data without requiring separate ethical approvals for each project. Ethical approval to collect participant data from the UK Biobank was provided by: the North West Multicenter Research Ethics Committee, which covers the UK; the National Information Governance Board for Health & Social Care, which covers England and Wales, and the Community Health Index Advisory Group, which covers Scotland.

## Results

### Baseline characteristics of the population

**Figure 1:**
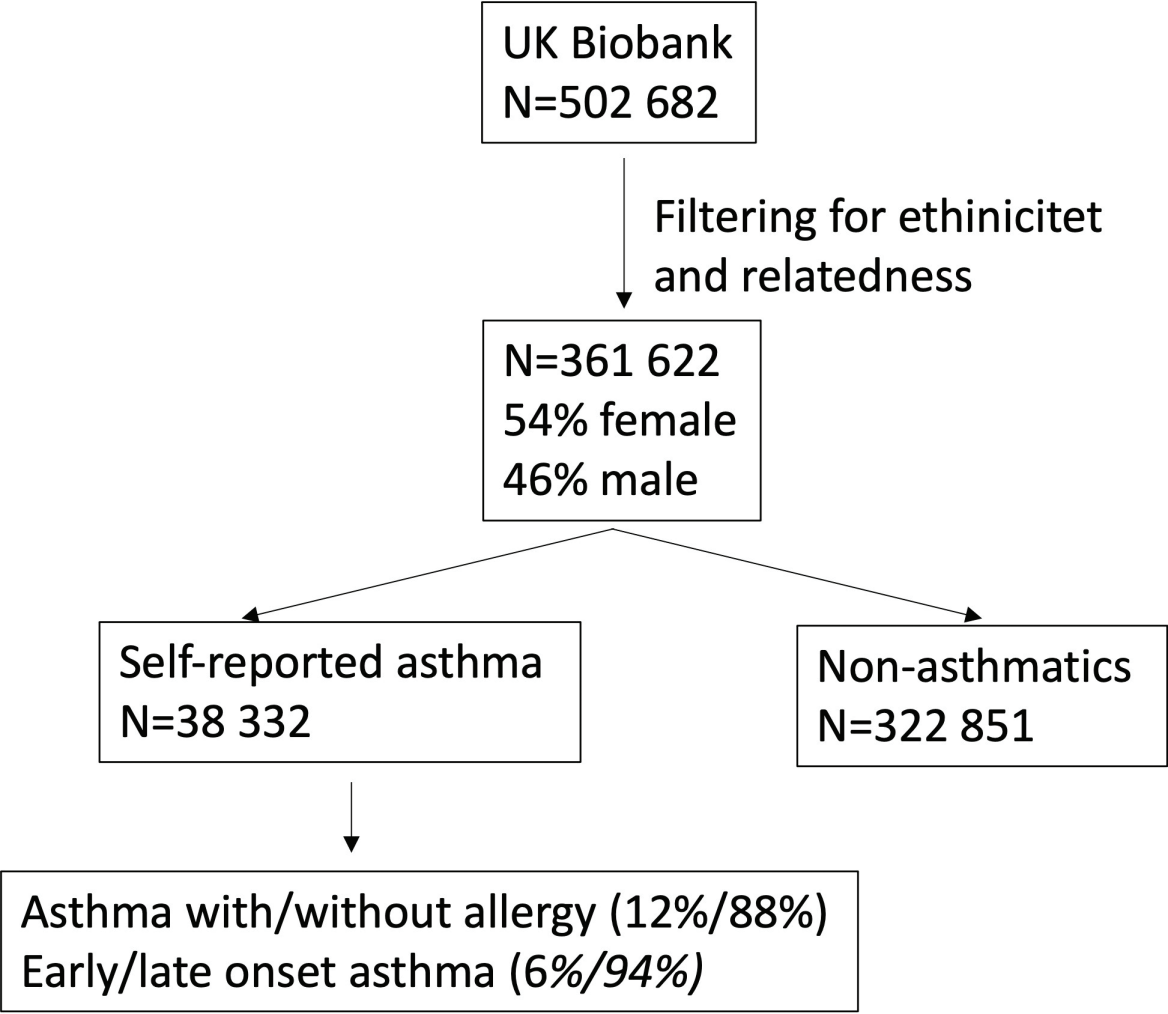
Flowchart describing the filtering of UKB participants and number of asthma- and asthma-subtype diagnosed participants.

A total of 361,622 participants were included in the study, of whom 54% were female. The inclusion process is illustrated in Figure 1. Participants were categorized into 38,332 individuals with self-reported doctor-diagnosed asthma and 322,851 non-asthmatic individuals.

Asthmatic participants were stratified into phenotypic subgroups, including asthma with or without allergy and early- or late-onset asthma, based on self-reported diagnosis details. The study population’s baseline characteristics are summarized in Table 1, whereas stratification by sex is presented in Supplementary Table 1.

**Table 1:** Baseline characteristics in the UK Biobank participants with and without asthma.

Characteristics	Asthma	No asthma
Total *n* (%)	38,332 (10.5)	322,851 (89.5)
Female, *n* (%)	21,968 **(**11.3**)**	172,456 **(**88.7**)**
Age (years), mean (SD)	55.85 (8.25)	57.0 (7.95)
BMI, mean (SD)	28.0 (5.1)	27.4 (4.6)
Smoking		
Ever (% of smokers)	2,271 (59.2)	19,419 (60.5)
Never (% of non-smokers)	1,549 (40.8)	12,762 (39.5)
Residential area population density		
The proportion of urban residences, *n* (%)	35,039 (91.4)	295,085 (90.2)
The proportion of rural residences, *n* (%)	2,923 (8.6)	24,751 (9.8)
Asthma phenotypes		
Early onset asthma (% of all asthmatics)	2,471 (6.4)	
Late onset asthma (% of all asthmatics)	3,587 (93.6)	
Asthma with allergy (% of all asthmatics)	4,586 (12.0)	
Asthma without allergy (% of all asthmatics)	3,375 (88.0)	

SD: standard deviation; BMI: body mass index.

### Residential area and asthma

In multivariable logistic regression models adjusted for age, BMI, and smoking status, residential area (rural vs. urban) was not significantly associated with asthma in either sex. Among females, the adjusted OR was 1.02 (95% CI: 0.97–1.08, *P* = 0.37), while in males, the adjusted OR was 1.03 (95% CI: 0.97–1.09, *P* = 0.31).

### Genotype distribution in asthmatics

The minor allele frequencies (MAF) for rs4986790 (Asp299Gly) and rs4986791 (Thr399Ile) were 5.9% and 6.3%, respectively. The two SNPs were in strong linkage disequilibrium (*R*² = 0.92). The distribution of these SNPs across urban and rural populations, stratified by sex, is presented in Supplementary Table 2.

### TLR4 polymorphisms in asthma and its phenotypes

In multivariable logistic regression models adjusted for age, BMI, smoking status, and 10 genetic principal components, there were no significant associations between rs4986790 (Asp299Gly) and rs4986791 (Thr399Ile) and asthma risk or between the SNPs and asthma phenotypes in either sex or the combined cohort (Table 2). Additionally, the effects of the SNPs were not modified by residential area population density in either males or females with asthma (Table 3) or across asthma phenotypes (Supplementary Table 3).

**Table 2:** Association between Asp299Gly and Thr399Ile and asthma or asthma phenotypes. It was calculated by logistic regression adjusted by age, BMI, smoking status, and 10 principal components to control for confounding effects and secondary interactions between covariates.

SNP	Combined cohort				Males				Females			
Asthma	*N* (cases + controls)	OR	95% CI	*P*	*N* (cases + controls)	OR	95% CI	*P*	*N* (cases + controls)	OR	95% CI	*P*
Asp299Gly	38,332 + 322,851	1.00	0.97–1.02	0.91	16,364 + 150,395	0.99	0.94–1.04	0.71	21,968 + 172,456	1.01	0.96–1.05	0.82
Thr399Ile	38,332 + 322,851	0.99	0.96–1.02	0.64	16,364 + 150,395	0.98	0.94–1.03	0.43	21,968 + 172,456	1.00	0.96–1.04	0.92
Asthma with allergy												
Asp299Gly	4,586 + 356,597	1.07	0.98–1.17	0.12	1,893 + 164,866	1.06	0.93–1.21	0.38	2,693 + 191,731	1.08	0.97–1.21	0.17
Thr399Ile	4,586 + 356,597	1.06	0.97–1.15	0.18	1,893 + 164,866	1.03	0.90–1.17	0.71	2,693 + 191,731	1.09	0.97–1.21	0.14
Asthma without allergy												
Asp299Gly	33,746 + 327,437	0.99	0.95–1.02	0.48	14,471 + 152,288	0.98	0.93–1.03	0.47	19,275 + 175,149	0.99	0.95–1.04	0.78
Thr399Ile	33,746 + 327,437	0.98	0.95–1.02	0.32	14,471 + 152,288	0.98	0.93–1.03	0.33	19,275 + 175,149	0.99	0.95–1.03	0.64
Early onset Asthma												
Asp299Gly	2,471 + 358,712	1.04	0.92–1.17	0.53	1,397 + 165,362	1.01	0.86–1.19	0.87	1,074 + 193,350	1.07	0.90–1.28	0.44
Thr399Ile	2,471 + 358,712	1.01	0.90–1.14	0.86	1,397 + 165,362	0.98	0.84–1.15	0.84	1,074 + 193,350	1.05	0.88–1.24	0.61
Late onset Asthma												
Asp299Gly	35,861 + 325,322	1.00	0.96–1.03	0.79	14,967 + 151,792	0.99	0.94–1.04	0.67	20,894 + 173,530	1.00	0.96–1.05	0.96
Thr399Ile	35,861 + 325,322	0.99	0.96–1.02	0.61	14,967 + 151,792	0.98	0.93–1.03	0.46	20,894 + 173,530	1.00	0.96–1.04	0.99

OR: odds ratio; CI: confidence interval.

**Table 3:** Interaction between rs4986790 (Asp299Gly) and rs4986791 (Thr399Ile) and residential area population density (RAPD), urban/rural, in males, females, or both sexes with asthma.

Group/genotype	Combined		
	β-estimate	95% CI	*P*
Asthma (*N*, cases/controls)	38,332/322,851		
rs489790:RAPD	0.033	−0.089 to 0.155	0.60
rs4986791:RAPD	0.042	−0.077 to 0.160	0.49
Males			
Asthma (*N*, cases/controls)	16,364/150,395		
rs489790:RAPD	0.081	−0.103 to 0.264	0.39
rs4986791:RAPD	0.075	−0.104 to 0.255	0.41
Females			
Asthma (*N*, cases/controls)	21,968/172,456		
rs489790:RAPD	0.001	−0.164 to 0.165	0.99
rs4986791:RAPD	0.021	−0.138 to 0.180	0.80

CI: confidence interval.

## Discussion

The main finding of this study is that TLR4 genetic variants (rs4986790/Asp299Gly and rs4986791/Thr399Ile) and residential area (urban vs. rural) were not associated with asthma susceptibility in British Caucasian participants of both sexes from the UK Biobank database. No significant associations were observed between TLR4 SNPs and asthma or asthma phenotypes (asthma with or without allergy, early – or late-onset asthma), nor between asthma and residential environment in either males or females.

To our knowledge, only one previous study has examined TLR4 variants, residential environment, and asthma interactions. Lau et al. reported that Asp299Gly and childhood rural living were not associated with early- or late-onset asthma in Tasmanian adults (28). However, direct comparisons with our study are challenging, as Lau et al. assessed residential area during childhood, whereas the UK Biobank only provides data on residential area at the time of study enrollment, involving middle-aged adults. Additionally, Lau et al.’s study did not evaluate Thr399Ile but instead investigated other Toll-like receptor (TLR) genes, including TLR6. Notably, two TLR6 SNPs (rs1039559 and rs5743810) were found to protect against early-onset asthma in individuals with childhood exposure to a farming environment (28).

Our study also found no association between TLR4 polymorphisms (Asp299Gly and Thr399Ile) and asthma, which is consistent with several earlier studies (19, 20, 29, 30). However, studies have reported associations between *TLR4* polymorphisms and asthma susceptibility (31, 32). A recent meta-analysis provided a more nuanced perspective and demonstrated that *Thr399Ile* (but not *Asp299Gly*) had a protective effect against asthma, but only in Asian populations, with no significant association observed in Caucasians, the population analyzed in our study (18). These findings suggest that genetic susceptibility to asthma linked to TLR4 polymorphisms may vary across ethnic groups, likely due to differences in genetic architecture, immune response, and environmental exposures.

Studies on ethnicity and genetic associations with asthma have highlighted ancestry-related differences in susceptibility. Research suggests that Asian populations often exhibit distinct asthma risk profiles compared to Caucasian populations, potentially due to genetic variations in immune response genes, environmental exposures, and lifestyle differences (14). The fact that our study focused exclusively on a British Caucasian population may partially explain the lack of association observed. In contrast, studies reporting significant associations may have included populations with different genetic backgrounds, influencing the observed effects. These findings emphasize the importance of considering ethnicity in genetic studies, as specific polymorphisms may have protective effects in some populations while increasing susceptibility in others (33).

The mechanisms underlying asthma susceptibility in the presence of TLR4 polymorphisms (Asp299Gly and Thr399Ile) have been linked to heightened responsiveness to LPS, a microbial component commonly found in farming and rural environments (15, 28). LPS recognition by TLR4 triggers downstream signaling, leading to the release of Th2 cytokines, which are implicated in asthma pathogenesis. Genetic alterations in innate immune pathogen recognition pathways may increase susceptibility to recurrent respiratory infections and atopy, potentially predisposing individuals to asthma (34). An elevated Th2 response has been associated with greater asthma risk (4). Other factors, including the age and duration of exposure to endotoxin, asthma phenotypes, genetic interactions, and environmental influences such as air pollution, may also contribute to modifying asthma susceptibility (35).

Our study found no significant association between residential area (urban vs. rural) and asthma susceptibility, which contrasts with previous research reporting higher asthma prevalence in urban environments. For instance, studies from Belgium have shown a greater proportion of asthmatics among adults living in urban areas compared to suburban settings (36, 37).

One possible explanation for these discrepancies is the variation in how urban and rural environments are defined across different studies, regions, and periods (38). Additionally, the dynamic nature of residential mobility may limit the ability to accurately capture long-term environmental exposures, potentially influencing the observed associations.

### Strengths and limitations

A major strength of this study is its use of a large, well-characterized cross-sectional cohort from the UK Biobank, which ensures sufficient statistical power for subgroup analyses across asthma phenotypes and sex. We stratified asthma by sex and clinically meaningful phenotypes (e.g. early/late onset and allergic/non-allergic asthma) to account for asthma’s heterogeneity. To minimize confounding genetic effects related to ethnicity, we restricted the analysis to British Caucasians. However, this choice is also limited, as the findings may not be generalizable to other ethnic groups. Additionally, while the UK Biobank had a low response rate (approximately 6% of invited participants enrolled), previous analyses have shown that risk factor associations in the UK Biobank correspond well with those in the general English population (39).

Another limitation of our study is that asthma and allergy data were self-reported, which may introduce recall bias and misclassification. Notably, the number of participants reporting allergic asthma was lower than expected, likely reflecting the characteristics of the UK Biobank cohort, which predominantly consists of adults and may underrepresent childhood-onset or allergy-associated phenotypes. Additionally, allergy was self-reported and may be subject to underreporting. The gold standard for allergy diagnosis involves specific immunoglobulin E (IgE) antibody testing (40), while asthma diagnosis in clinical settings typically relies on spirometry to confirm reversible airflow limitation. However, these objective diagnostic measures were not available in the UK Biobank at the time of this study.

Furthermore, the study lacked an objective marker of microbial exposure, such as endotoxin levels, which limits the ability to fully assess the relationship between TLR4 SNPs, microbial exposure, and asthma risk. Instead, rural residence was used as a proxy for microbial exposure, which may not accurately capture individual variation in environmental microbial exposure. In addition, information on place of birth or other indicators of early-life environmental exposures is not currently available via the UK Biobank.

An additional limitation of our study is its focus on a single gene variant. While this allowed for a more detailed investigation of its functional relevance and potential interaction with environmental exposures, it also constrained the scope of genetic discovery. In contrast, genome-wide association studies (GWAS) can identify novel loci but often lack biological specificity and typically require larger sample sizes and stricter multiple testing corrections (41). Our models did not include gene–gene interactions, as their reliable detection demands more complex analytical frameworks and substantially larger datasets. However, we acknowledge that excluding such interactions may overlook subtle modifying effects and consider this an important direction for future research.

While a case–control design can be useful for rare outcomes, we used a population-based cohort to enable estimation of prevalence and population-level interactions. Although the cohort design does not address class imbalance, many asthma cases (*n* > 38,000) mitigated this issue for main effects. However, some interaction strata may still have limited numbers, which potentially reduces the power of the detection of interaction effects.

In addition, we employed logistic regression models in this study as they are the standard approach for binary outcomes and allow for interpretability across subgroups. This method assumes symmetric odds and may not perfectly capture disease processes like asthma, particularly in relation to continuous variables such as age. In this analysis, we did not test non-symmetric binary regression models (e.g. complementary log-log models), which could be assessed in future studies, particularly when investigating sex-related effects.

Another weakness is that we used the same covariates: age at recruitment, BMI, and smoking status for both early- and late-onset asthma models. These covariates reflect current life characteristics and may not accurately represent exposures or physiological status at the time of asthma onset in early childhood. In further research, other models may be more appropriate for understanding associations rather than inferring causal pathways, particularly for early-onset asthma.

In our study, we did not use spatial correlation as we could not access finer-grained spatial coordinates that would enable modeling spatial correlation directly (e.g. via spatial regression or geostatistical methods). However, we used residential area (urban vs. rural) as a proxy for environmental exposure, and this was treated as a binary variable derived from population density data. Including spatial correlation in the models increases the risk of overfitting and further inflates the *P*-values in a non-significant context.

Given our large sample size (>360,000 participants), including over 38,000 asthma cases, the study had sufficient power to detect modest associations (e.g. OR of 1.10 or smaller) with 80–90% power at α = 0.05, depending on the specific analysis. However, the power may be lower for some phenotypes, such as early-onset or allergic asthma, especially when stratified by sex or residential area. The number of cases in these subgroups was more limited, which may reduce the power to detect minor interaction effects. However, as shown by Majumdar et al., statistical power in gene–environment studies can be further improved using multivariate approaches and two-step testing strategies, especially in the presence of pleiotropy (42). While our research focused on univariate models, future extensions could incorporate multivariate phenotypes or implement a two-step framework to increase sensitivity for detecting gene-environmental interactions.

### Clinical relevance and future directions

Understanding the genetic and environmental interactions in asthma susceptibility is crucial for developing targeted preventive and therapeutic strategies (4, 11). Although our study found no association between *TLR4* polymorphisms, residential area, and asthma, previous research suggests that genetic risk factors may vary across ethnic groups (14). This highlights the need for personalized approaches in asthma management based on genetic background and environmental exposures (28).

Future studies should explore the potential protective effects of *TLR4* variants in different populations, particularly among Asians, where some associations have been observed. Additionally, given the influence of microbial exposure on immune system development (8), further research should incorporate more precise measures of environmental factors, such as endotoxin levels and long-term residential mobility data, rather than relying solely on urban-rural classifications.

Expanding studies to include multi-ethnic cohorts and integrating objective asthma diagnostic markers, such as spirometry and IgE measurements, will enhance the accuracy of findings. Investigating other genes may also provide new insights into the genetic regulation of asthma susceptibility (35).

Finally, a deeper examination of urbanization-related factors, such as air pollution, diet, and occupational exposure, could further elucidate environmental contributors to asthma risk (33). Addressing these gaps will advance our understanding of gene-environment interactions in asthma and contribute to more effective, personalized prevention and treatment strategies.

## Conclusions

In conclusion, our study found no statistically significant association between *TLR4* polymorphisms (*Asp299Gly* and *Thr399Ile*) and asthma, regardless of sex or residential area. Considering our findings in the context of previous research, it is evident that the interplay between environmental exposures, gender, and genetic susceptibility in asthma pathophysiology is highly complex.

The role of ethnicity in these interactions should be further explored, as emerging evidence suggests that genetic associations with asthma risk may vary across populations. Our findings also highlight the need for a broader examination of factors related to urbanization, such as air pollution and household chemicals, which may also contribute to asthma development. Expanding studies to include multi-ethnic cohorts and incorporating more precise genetic and environmental measures will be essential for improving our understanding of the intricate gene–environment interactions underlying asthma susceptibility.

## Author contributions

Conceptualization, ARA, MAK; methodology, ARA, MRA; software, MRA; validation, all authors; investigation: MAK, MRA; resources, ARA; data curation, MR, MAK; writing – original draft preparation, MAK; writing – review and editing, all authors; visualization, MAK, MRA; supervision, ARA, ÅJ, WEE; project administration, MAK; funding acquisition, ARA. All authors have read and agreed to the published version of the manuscript.

## Data availability statement

The data used in the present study are available for research purposes after ethical approval at https://www.ukbiobank.ac.uk/.

## Disclosure statement

The authors declare no conflicts of interest.

## Supplementary Material


