# Supplementary material for: Toll-like receptor 4 (TLR-4) polymorphisms and asthma risk in rural and urban settings: findings from the UK biobank
Source: Ups J Med Sci. 2025 Jul 4;130:10.48101/ujms.v130.12243. doi: 10.48101/ujms.v130.12243 (PMC12320926; doi:10.48101/ujms.v130.12243)
Supplement: Supplementary file 1 [file UJMS-130-12243-s1.pdf]

Supplementary materials

Supplementary table 1: Baseline characteristics in the UK Biobank participants divided into asthma and no asthma, and stratified by sex.

	Asthma		No asthma	
	**Female**	**Male**	**Female**	**Male**
**n (%)**	21,968 (11.3%)	16,364 (9.8%)	172,456 (88.7%)	150,395 (90.2%)
**Age (y), mean (SD)**	55.9 (8.1)	55.8 (8.4)	56.8 (7.9)	57.3 (8.0)
**BMI, mean (SD)**	28.0 (5.7)	28.0 (4.4)	26.9 (5.0)	27.8 (4.2)
**Smoking**				
**Ever (% of smokers)**	12,278 (56.1%)	10.430 (64.0%)	95, 909 (55.8%)	98,276 (65.6%)
**Never (% of non-smokers)**	9607 (43.9%)	5878 (36%)	76,000 (44.2%)	51,618 (34.4%)
**Residential area population density**				
**The proportion of urban residences**	20,097 (11.3%)	14,942 (9.8%)	157,576 (88.7%)	137,509 (90.2%)
**The proportion of rural residences**	1680 (11.1%)	1243 (9.9%)	13,397 (88.9%)	11,354 (90.1%)
**Asthma phenotypes**				
**Early onset asthma (% of all asthmatics)**	1074 (4.9%)	1397 (8.5%)	x	x
**Late onset asthma (% of all asthmatics)**	20894 (95.1%)	14976 (91.5%)	x	x
**Asthma with allergy (% of all asthmatics)**	2693 (12.3%)	1893 (11.6%)	x	x
**Asthma without allergy (% of all asthmatics)**	19275 (87.7%)	14471 (88.4%)	x	x

Supplementary Table 2: Genotype distribution of rs4986790 (Asp299Gly) and rs4986791 (Thr399Ile) for asthma and asthma phenotypes in male and female participants.

	Urban		Rural	
**rs4986790 (AA/AG/GG)**	**Female**	**Male**	**Female**	**Male**
**Asthma**	17781/2242/74	13,269/1607/66	1498/177/5	1102/134/39
**No asthma**	139,320/17,692/564	121,726/15,270/513	11,934/1424/39	1102/134/7
**Early onset asthma**	971/135/4	1323/157/7	105/12/0	131/14/0
**Late onset asthma**	16941/2125/72	12150/1470/61	1403/167/5	993/121/7
**Asthma with allergy**	2139/287/7	1494/195/7	203/28/0	158/19/0
**Asthma without allergy**	15642/1955/67	11775/1412/59	1295/149/5	944/115/7
**rs4986791 (CC/CT/TT)**				
**Asthma**	17,652/2363/82	13,192/1676/74	1484/190/6	1095/141/7
**No asthma**	138,246/18,702/628	120,799/16,149/561	11,842/1514/41	10,041/1268/45
**Early onset asthma**	968/138/4	1318/162/7	102/15/0	131/14/0
**Late onset asthma**	16814/2244/80	12078/1534/69	1392/177/6	986/128/7
**Asthma with allergy**	2124/300/9	1490/197/9	198/33/0	158/19/0
**Asthma without allergy**	15528/2063/73	11702/1479/65	1286/157/6	937/122/7

Supplementary Table 3. Interaction between rs4986790 (Asp299Gly) and rs4986791 (Thr399Ile) and residential area population density (RAPD), urban/rural, in males or females or both sexes with allergic asthma and early onset asthma.

	Combined			Males			Females		
	**β-estimate**	**95% CI**	**P**	**β- estimate**	**95% CI**	**P**	**β-estimate**	**95% CI**	**P**
**Asthma with allergy (N, cases/controls)**	4586/356,597			1893/164,866			2693/191,731		
**rs489790:RAPD**	-0.057	-0.372-0.258	0.72	-0.132	-0.622-0.357	0.60	-0.012	-0.427-0.402	0.95
**rs4986791:RAPD**	0.005	-0.296-0.306	0.97	-0.170	-0.659-0.319	0.50	0.117	-0.268-0.503	0.55
**Non-allergic asthma (N, cases/controls)**	33,746/327,437			14,471/152,288			19,275/175,149		
**rs489790:RAPD**	0.043	-0.088-0.173	0.52	0.113	-0.081-0.308	0.25	-0.009	-0.185-0.166	0.92
**rs4986791:RAPD**	0.043	-0.084-0.170	0.51	0.114	-0.076-0.303	0.24	-0.008	-0.179-0.163	0.92
**Early onset asthma (N, cases/controls)**	2471/358,712			1397/165,362			1074/193,350		
**rs489790:RAPD**	-0.164	-0.604-0.277	0.47	-0.099	-0.688-0.489	0.74	-0.242	-0.913-0.429	0.48
**rs4986791:RAPD**	-0.066	-0.485-0.352	0.76	-0.140	-0.729-0.448	0.64	0.024	-0.577-0.626	0.94
**Late onset asthma (N, cases/controls)**	325,322/35,861			14,967/151,792			20,894/173,530		
**rs489790:RAPD**	0.051	-0.075-0.176	0.43	0.095	-0.095-0.286	0.33	0.018	-0.50-0.186	0.84
**rs4986791:RAPD**	0.052	-0.071-0.175	0.40	0.095	-0.092-0.281	0.32	0.022	-0.141-0.185	0.79
